# Smartphone IMU Sensors for Human Identification through Hip Joint Angle Analysis

**DOI:** 10.3390/s24154769

**Published:** 2024-07-23

**Authors:** Rabé Andersson, Javier Bermejo-García, Rafael Agujetas, Mikael Cronhjort, José Chilo

**Affiliations:** 1 Department of Electrical Engineering, Mathematics and Science, University of Gävle, 801 76 Gävle, Sweden; milcrt@hig.se (M.C.); jco@hig.se (J.C.); 2 Departamento de Ingeniería Mecánica, Energética y de los Materiales, Escuela de Ingenierías Industriales, Universidad de Extremadura, 06006 Badajoz, Spain; javierbg@unex.es (J.B.-G.); rao@unex.es (R.A.)

**Keywords:** smartphone sensors, IMU sensors, person recognition, machine learning classification, human motion analysis

## Abstract

Gait monitoring using hip joint angles offers a promising approach for person identification, leveraging the capabilities of smartphone inertial measurement units (IMUs). This study investigates the use of smartphone IMUs to extract hip joint angles for distinguishing individuals based on their gait patterns. The data were collected from 10 healthy subjects (8 males, 2 females) walking on a treadmill at 4 km/h for 10 min. A sensor fusion technique that combined accelerometer, gyroscope, and magnetometer data was used to derive meaningful hip joint angles. We employed various machine learning algorithms within the WEKA environment to classify subjects based on their hip joint pattern and achieved a classification accuracy of 88.9%. Our findings demonstrate the feasibility of using hip joint angles for person identification, providing a baseline for future research in gait analysis for biometric applications. This work underscores the potential of smartphone-based gait analysis in personal identification systems.

## 1. Introduction

The past few decades have witnessed unprecedented advancements in smartphone technologies. Each year, these handheld devices, which are equipped with a diverse array of sensors, have grown more sophisticated [1]. These sensors serve various functions, from accelerometers, magnetometers, and gyroscopes to environmental sensors like ambient light and temperature sensors [2,3].

Smartphone sensors have already found valuable applications across multiple fields, including health and rehabilitation, fitness by tracking physical activities, monitoring heart rates, and measuring sleep patterns [4,5]. In the automotive industry, they are employed for vehicle navigation and accident detection [6]. Environmental monitoring and augmented reality also benefit from the data collection and processing capabilities of smartphone sensors [7]. Building upon these technological advancements, smartphones have also emerged as a robust means of human identification through gait recognition [8,9,10].

Gait monitoring using sensory data has gained increased research attention over the years as gait biometrics is a unique pattern of human locomotion. Every locomotion pattern is unique due to variations in magnitude and time among people but identifiable as it is performed naturally and habitually every day, which involves many muscles and joints [8]. Similar to other biometric data such as iris, voice samples, and fingerprints, gait analysis has appeared in applications for security and identification purposes. Unlike other biometric data, gait patterns have a multidimensional and convoluted nature, making them extremely difficult to mimic or steal, just like fingerprints or voice patterns [11].

Gait analysis can be captured by foot pressure sensors, wearable sensors, or a vision-based system using multiple cameras or stereo vision [12,13]. However, foot pressure sensors and vision-based systems require a spacious work area, which is costly and restricted to laboratory research that depends on complex and multi-sensor settings and specialized personnel to carry out the operations, which make them impractical for many applications [14,15]. Therefore, the integration of smartphone sensors for gait analysis and person recognition offers numerous potential benefits [16,17,18]. First, the availability and affordability of smartphone sensors make them accessible to a broad demographic; in fact, 85.74% of the world population used smartphones in the year 2023, as illustrated in [19]. Second, their portability and user-friendly interfaces render them ideal for delivering gait recognition in various settings, from hospitals to home environments [20].

Numerous studies have investigated person recognition through gait analysis, employing various techniques and sensor placements. For example, Hoang et al. demonstrated the feasibility of gait recognition using smartphone accelerometers, achieving up to 92.7% accuracy [21]. Connor and Ross provided a comprehensive survey on gait recognition modalities, highlighting the effectiveness of full-body motion capture and foot pressure patterns [12]. Makihara et al. discussed the use of multiple joints and their combined movement patterns for accurate identification [22]. While these studies used multiple sensor inputs, our focus on hip movement is justified by the work of Derawi et al., which showed that hip rotation patterns are highly individualistic and can be effectively captured by waist-mounted accelerometers. Concentrating on hip movement reduces the impact of variations in arm swing or upper body movements, making this approach more user-friendly [23].

Hip joint analysis offers several advantages: it is central to gait biomechanics as the primary connection between the lower limbs and the trunk [24]; requires fewer sensors than full-body analysis [25]; preserves privacy better than facial recognition or full-body gait analysis [26]; is sensitive to pathological, neurological, and musculoskeletal conditions [27]; applies directly to many rehabilitation protocols [28]; and correlates strongly with energy expenditure during walking, as 45% of the mechanical energy comes from the hip joint [29,30]. These benefits, combined with smartphone ubiquity, make hip joint analysis a promising approach to study for gait movement monitoring and person recognition.

However, gait movement data can be hard to interpret or analyze [15,31]; thus, researchers use machine learning techniques for gait recognition and data analysis captured by a camera, force-based systems, or force-sensitive resistors. For this reason, multiple machine learning algorithms, such as support vector machine (SVM), neural networks (NNs), long short-term memory neural network (LSTM NN), recurrent neural network (RNN), naive Bayes (NB), linear discriminant analysis (LDA), and hybrid convolutional neural network (HCNN), were utilized in previous studies for gait recognition and classification to extract a comprehensive understanding of biometric data based on human movements [9,32,33]. Many ML techniques showed their superior performance and powerful use in various fields, including human recognition, manufacturing, robotics, quality inspection, sports performance analysis, and medical diagnosis [31,34].

Therefore, this study explores the use of smartphone IMU sensor data with ML classification techniques, including perceptron, logistic regression, nearest neighbor rule, naive Bayes, and random forests, to recognize persons based on their hip joint angle. We validate smartphone gait movement by comparing it with MCSs using a pendulum test bench. Our future goal is to utilize this system identification approach in a rehabilitation hip joint exoskeleton system within a healthcare environment where data can be anonymously shared with a healthcare system to recognize patients before giving or following their treatments to avoid sharing any sensitive personal information.

The paper is structured as follows: Section 2 describes the theory behind the steps conducted in the study, while Section 3 discusses the methods used throughout this research, with subsections that shed light on the measurement comparison, the test bench with the MCS and smartphone configuration, and the machine learning analysis. Section 4 is devoted to presenting the results and discussion. Finally, Section 5 concludes with the paper’s findings and analysis.

## 2. Theory

To conduct a hip joint analysis using smartphone sensors, we elucidate the backbone theoretical key of our research, beginning with the reason behind human gait analysis, followed by the role of inertial measurement units (IMUs) in smartphones and some signal-processing techniques used.

### 2.1. Human Gait Analysis

Human walking is fundamentally a periodic activity characterized by repetitive motions of body segments. Human gait analysis is pivotal in rehabilitation for the precise identification of walking abnormalities and biomechanical inefficiencies. By analyzing gait patterns, clinicians can tailor rehabilitation programs to address specific deficits, thereby enhancing the efficacy of interventions and improving patient outcomes or combining it with rehabilitation robotic exoskeleton sessions [35]. Since exoskeleton devices comprise sensors, actuators, and electronic circuits operating in close contact with patients, understanding the intricacies of gait mechanics is pivotal for ensuring that these devices operate safely and reliably, identifying walking abnormalities for orthotics or prosthetic leg users, and optimizing movements to mitigate posture-related issues [30].

Gait analysis can include kinematics, kinetics, or EMG measurements. Gait kinematics describes the motion of the major joints and segments of the lower extremity, such as the hip, knee, ankle, and foot, while gait kinetics studies the forces that result from the movement of human gait segments, including the ground reaction force, joint reaction force, and joint torqueEMG sensors, to measure the electrical activity of the muscles that control the movement of these segments, such as the quadriceps, hamstrings, gastrocnemius, and tibialis anterior [35]. However, among these techniques, the measurement of gait kinematics is pivotal for recognizing the gait phases, joint angles, and segment movements.

To systematically evaluate human gait in a kinematics-based manner, an MCS, with the use of computer vision techniques, stands out for its unparalleled accuracy, utilizing either marker-based or markerless methods to achieve results precise to within 1 mm [36]. These systems use reflective markers placed on the person’s body or markerless image processing techniques to capture human gait. However, these systems are expensive and out of reach for many clinicians, especially in developing countries [37].

Thus, alternative kinematic measurement techniques can be performed using wearable sensors such as inertial measurement units (IMUs) or magnetic, angular rate, and gravity (MARG) sensors fused in micro-electro-mechanical systems (MEMSs) in smartphones for indoor and outdoor measurements, in contrast with MCSs for laboratories.

### 2.2. IMUs and MARG in Smartphones

**Figure 1:**
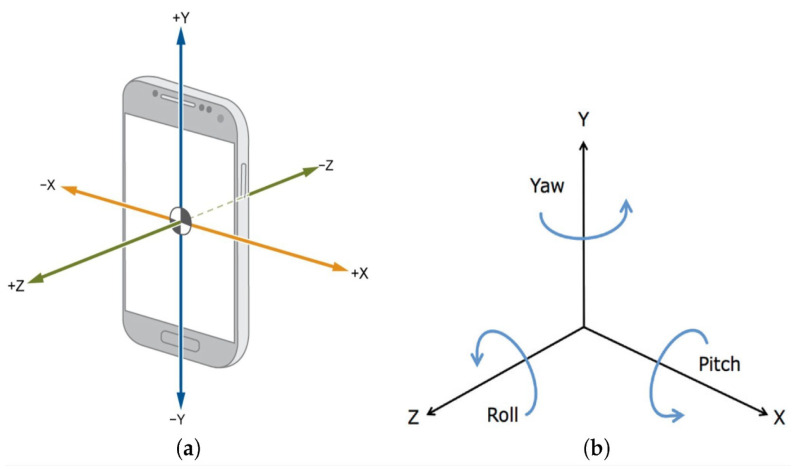
(**a**) The three-axial sensor measurements of the smartphone; (**b**) the fixed reference frame of the earth [40].

Smartphones have multiple embedded motion sensors, such as accelerometers, gyroscopes, and magnetometers, that can offer alternative solutions compared with external IMUs for gait recognition. In addition, IMUs and MARG can possess multiple advantages, such as low power consumption, lightweight, and ease of use [38,39]. These sensors are three-axial sensors that capture the acceleration $a_{i}$ along the X, Y, and Z axes corresponding to the roll (ϕ), pitch (θ), and yaw (ψ) axes, as shown in Figure 1 and represented in (1).

(1) $$a_{i}={\left[\begin{array}{ccc} a_{x} & a_{y} & a_{z} \end{array}\right]}^{T}$$

The gyroscope and magnetometer are also three-axial sensors. The accelerometer gauges three-axial linear acceleration ($a_{i}$), the gyroscope quantifies angular velocity ($\omega_{i}$), and the magnetometer assesses the magnetic field ($m_{i}$). These sensors’ data can be used to analyze human gait movements and to find the joint trajectories by fusing them to obtain the orientation angles [35].

### 2.3. The Sensor Fusion and Signal Processing

The sensor measurements of MARG and IMU usually have intrinsic drift and high-degree noise, making it challenging to reconstruct trajectories and orientation estimation directly [41]. Therefore, finding the orientation estimation of the smartphone (ϕ, θ, ψ) based only on angular velocities (ω) from a gyroscope sensor gives inaccurate measurements, as they include bias ($b_{\omega }$) (low-frequency noise) and Gaussian noise ($W_{\mathrm{Noise}}$) associated with the true angular velocity ($\omega_{\mathrm{true}}$). However, integrating any error from the gyroscope leads to a drift in orientation estimation over time due to the integration operation. Similarly, using only accelerometer measurements (*a*) is an inadequate method for orientation estimation as its measurements include bias ($b_{a}$), noise ($W_{\mathrm{Noise}}$), gravitational acceleration ($a_{\mathrm{gravity}}$), and non-gravitation acceleration ($a_{\mathrm{true}}$), which consequently lead to estimation with high-frequency noise. Thus, accumulated errors caused by integration calculations of gyroscope measurements can be solved by fusing measurements provided by an accelerometer and magnetometer in one of the sensor fusion techniques [38,42]. However, the magnetometer measurements (*m*) suffer from the influence of magnetic field interference ($m_{\mathrm{init}}$), bias ($b_{m}$), and noise ($W_{\mathrm{Noise}}$) in addition to the true magnetometer measurement ($m_{\mathrm{true}}$), as shown in Equations (2)–(4) [43].

(2) $$\omega =\omega_{\mathrm{true}\;}+b_{\omega }+W_{\mathrm{Noise}},$$

(3) $$a=a_{\mathrm{true}\;}+a_{\mathrm{gravity}\;}+b_{a}+W_{\mathrm{Noise}},$$

(4) $$m=m_{\mathrm{true}\;}+m_{\mathrm{init}\;}+b_{m}+W_{\mathrm{Noise}}.$$

Therefore, the sensor fusion technique is an essential procedure to determine the accurate orientation. This technique combines data from multiple sensors to provide a more reliable and comprehensive understanding of drift-free and noise-free spatial orientation [37].

In the literature, sensor fusion algorithms (SFAs) are predominantly categorized into deterministic and stochastic frameworks. Within the deterministic paradigm, algorithms such as linear complementary filters (LCFs) and nonlinear complementary filters (NCFs) are commonly employed. In contrast, the stochastic domain encompasses a diverse array of algorithms, including linear Kalman filters (LKFs), extended Kalman filters (EKFs), complementary Kalman filters (CKFs), square root unscented Kalman filters (SRUKFs), and square root cubature Kalman filters (SRCKFs) [44].

It is noteworthy that LKF has been utilized for orientation estimation in MARG and IMU sensor arrays, but it has limitations in adequately addressing the inherent non-linearities present in real-time systems [37]. Thus, many studies have proposed advanced SFAs such as the EKF used in the attitude heading reference system (AHRS) [45].

**Figure 2:**
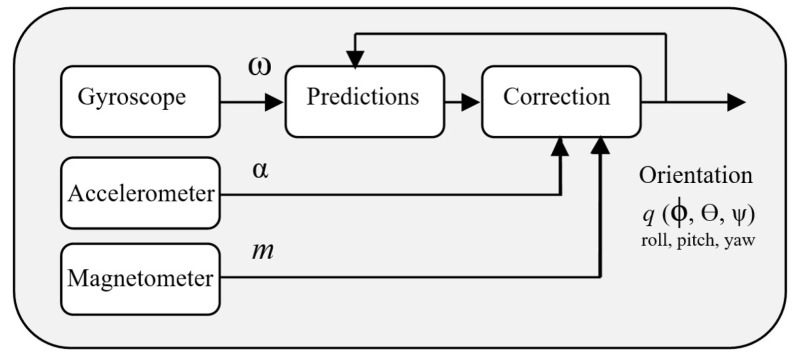
The attitude heading reference system (AHRS) algorithm.

AHRS is a particularly indirect EKF quaternion-based algorithm that has the ability to estimate magnetic disturbances, which can mitigate the effect of interference $m_{\mathrm{init}\;}$ and makes it useful for its robustness in various applications [42]. It consists of a two-step process: prediction and correction to determine the orientation *q* (ϕ, θ, ψ), as illustrated in [46] and shown in Figure 2 [47]. The prediction phase relies on gyroscope data based on integrating the angular velocity (*w*), while the correction phase utilizes acceleration signals (*a*) and magnetometer readings (*m*). The AHRS algorithm updates the orientation estimation *q* by comparing it with the orientation in the prediction phase to minimize the error between the estimated and the actual orientation through iterative correction procedures based on the accelerometer and the magnetometer [48].

## 3. Methods

### 3.1. Participants

In this paper, 10 subjects (8 males and 2 females) were asked to walk on a treadmill (BH F2W Dual) for 10 min at a constant normal walking speed of 4 km/h. Summary statistics (mean ± standard deviation (SD)) included age (33.7±7.65) ranging from 22 to 45 years, weight in kg (63.75±10.33), height in m (1.74±0.09), and body mass index (kg/m^2^): (21.75±2.24).

The subjects were in good health and free of any visible walking impairments. All subjects were instructed to participate in a preliminary warm-up session for 5 min of walking on a treadmill to guarantee the safety of the participants and familiarize them with the treadmill environment before the experimental measurements. All experimental procedures were approved by the Local Ethics Committee at the University of Extremadura.

### 3.2. Comparison of Measurements

**Figure 3:**
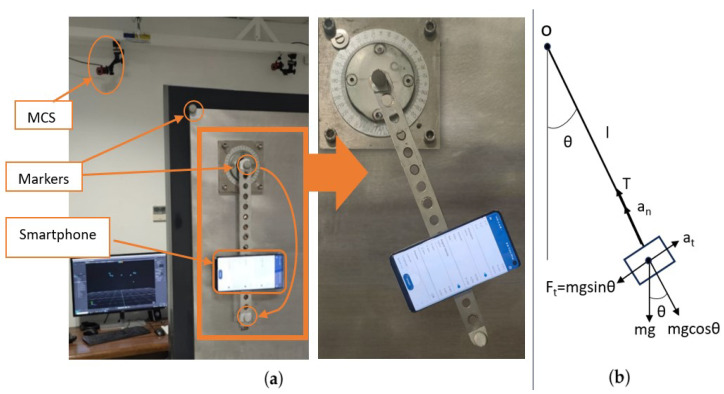
(**a**) The test bench configuration to compare the MCS and the smartphone measurements; (**b**) the theoretical sketch of the configuration of a pendulum.

To compare the angle estimation using a smartphone with a motion capture system (MCS), a pendulum was set on a kinematic test bench, located in the mechanical engineering laboratory at the University of Extremadura, as shown in Figure 3a. It consisted of a smartphone placed at one end of a link, while the other end articulated at a fixed point (referred to as point O), allowing it to rotate 360°. This same point coincided with the center of a goniometer, enabling the user to select the initial amplitude ($\theta_{0}$) at which the oscillation began at time t = 0.

The idea was to compare the angle measurements of a pendulum using a smartphone and an MCS. The MCS consisted of 8 cameras (Optitrack, Natural Point, Corvallis, OR, USA). The cameras’ frame rate was set at 100 Hz. Before starting the recordings, a calibration of the space was performed using a calibration square on the floor and a T-wand. To measure the angle, three markers were placed on the test bench, one on the test bench corner, another marker at the tip of the pendulum axis, and the last one at the base of the pendulum. Figure 3a shows the configuration of the camera system and the position of the markers on the test bench for the pendulum measurements.

The comparison was conducted by moving the pendulum at small angles (around 10°). Given the forces acting on a pendulum as shown in Figure 3b, the pendulum angle can also be theoretically calculated from the differential equation, which is

(5) $$\ddot{\theta }+\frac{g}{l}\theta =0,$$

where $\ddot{\theta }$ represents the pendulum’s angular acceleration, *g* is the acceleration of gravity, *l* is the length of the pendulum, and θ is the angular position.

Consider the equation for the simple harmonic motion,

(6) θ(t)=Asinωt+ϕ,

where *A* is the amplitude of motion, ω denotes the angular velocity, *t* is the time variable, and ϕ is the phase angle. Additionally, it is noted that the term (g/l) multiplied by θ in Equation (5) represents the square of the angular velocity, denoted as $\omega^{2}$. Therefore, for t=0 and small angles (around 10°), the equations that describe the kinematics of the pendulum are

(7) $$\theta \left(t\right)=\theta_{0}\sin\left(\omega t+\frac{\pi }{2}\right),$$

(8) $$\dot{\theta }\left(t\right)=\omega \theta_{0}\cos\left(\omega t+\frac{\pi }{2}\right),$$

(9) $$\ddot{\theta }\left(t\right)=-\omega^{2}\theta_{0}\sin\left(\omega t+\frac{\pi }{2}\right),$$

where θ(t), $\dot{\theta }\left(t\right)$, and $\ddot{\theta }\left(t\right)$ are the angular position, angular velocity, and angular acceleration, all three depending on time and the initial angular position $\theta_{0}$.

### 3.3. Hip Joint Identification

The identification of hip joint angles was conducted in two parts: data acquisition and feature extraction and ML classification techniques.

#### 3.3.1. Data Acquisition

**Figure 4:**
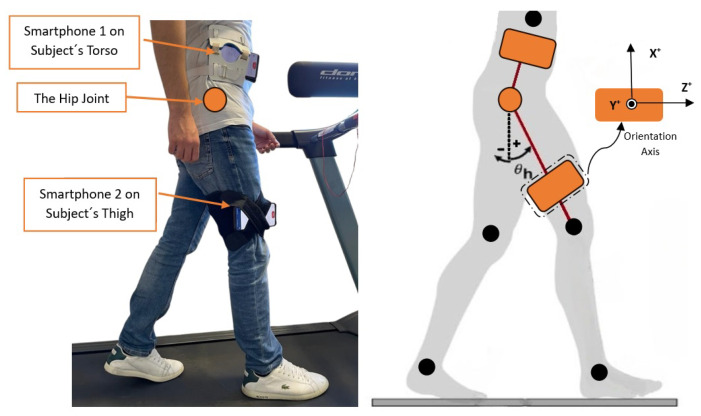
The smartphone setup on a subject during the test.

For this study, 2 smartphones were used to measure the hip joint angle based on 3-axial measurement (X, Y, and Z) coordinates: the acceleration in meters per second squared (m/s^2^), the angular velocity in radians per second (rad/s), and the magnetic field in microteslas (μT). One smartphone was mounted on the subject’s torso, while another smartphone was attached to the thigh like a pendulum-like structure, as shown in Figure 4. The sampling rate for recording the data was 100 Hz.

After mounting the smartphones on the subject’s torso and thigh, an initial calibration was conducted, where the subject was standing in an upright position for 5 s. This calibration is needed for high-accuracy measurements as MEMS sensors are manufacturer-calibrated, but some errors can arise over time [43]. In addition to the calibration procedure and to mitigate the impact of wearing error, we implemented a standardized protocol for smartphone placement to minimize variability. The device was securely fastened to the lateral side of the thigh and torso using a specially designed adjustable strap, ensuring consistent positioning across participants. This approach is supported by Jayasinghe et al., who demonstrated a strong correlation (above 0.9) between loose clothing-mounted sensors and body-mounted sensors when placed on the thigh and shank. This correlation indicates that our methodology of placing smartphones on the thigh is robust against variations in wearing conditions, thus minimizing potential biases [49].

**Figure 5:**
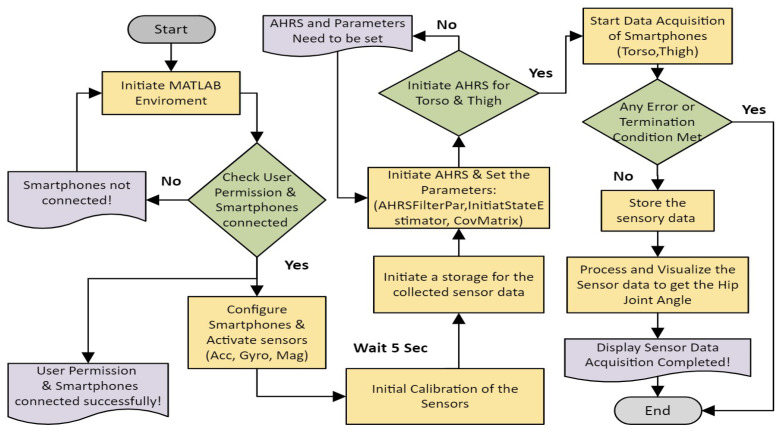
The flowchart for acquiring and processing sensor data.

Then, with the sensor fusion technique, the sensors provide Euler angles $\phi_{1}$, $\theta_{1}$, and $\psi_{1}$ for the torso from the orientation around X, Y, and Z of smartphone 1 and $\phi_{2}$, $\theta_{2}$, and $\psi_{2}$ for the thigh, which come from the orientation around X, Y, and Z of smartphone 2. Therefore, the relative hip joint angle ($\phi_{h}$, $\theta_{h}$, $\psi_{h}$) was calculated as the difference between the torso and thigh angles, as illustrated in Figure 4 and the following equations:

(10) $$\phi_{h}=\phi_{2}-\phi_{1},$$

(11) $$\theta_{h}=\theta_{2}-\theta_{1},$$

(12) $$\psi_{h}=\psi_{2}-\psi_{1},$$

where $\phi_{h}$ is the roll angle that represents the abduction/adduction of the hip joint, while $\theta_{h}$ represents the flexion/extension and $\psi_{h}$ represents the internal/external rotation because the hip joint is mechanically represented as a ball-and-socket joint. However, our angle of interest for this study was the hip joint flexion/extension, as shown in Figure 4, as the data serve a planned hip rehabilitation exoskeleton moving in a sagittal plane like the one shown in the research [30,50]. The functions to connect the smartphones with MATLAB version R2024a in the cloud and the AHRS filters were called using MATLAB functions and the “sensor fusion and tracking” toolbox, as illustrated in [51,52]. Therefore, the flowchart for acquiring and processing data from two smartphones is illustrated in Figure 5, which was the pre-stage for feature extraction and classification techniques.

#### 3.3.2. Feature Extraction and Classification Techniques

For this study, the duration of data acquisition for each participant was consistently fixed at 600 s, which was divided into 100 intervals, with each interval lasting 6 s. Each interval was considered a separate walking trial. Therefore, 100 trials were used to capture the normal walking patterns of each participant to facilitate the feature extraction process and, subsequently, the training models using ML. Furthermore, 10 classes were assigned to represent 10 subjects, and each class contained 100 feature vectors.

As people have various walking styles, we employed statistical calculations focusing on the angles of the hip joint—expressed in degrees, where 0 degrees represent the case when the subject is in an upright position and positive and negative degrees represent flexion and extension, respectively, as shown in Figure 4 [53]. Time domain features are extensively used in biological systems due to their lower computational complexity and ease of implementation [54]. This led to extracting nine time-domain distinct features of each trial per subject (class). The features capture the dynamic characteristics of hip trajectory, providing critical insights into the variability and overall patterns of movement. The extracted features were mean value (MV), median (M), maximum angle, covariance (COV), minimum angle, variance (VAR), standard deviation (SD), kurtosis (KUR), and skewness (SKE) of 100 datasets for each subject. The detailed calculations of nine extracted features are shown in Table 1 and discussed in [40,55].

For hip joint angle analysis and classification, principal component analysis (PCA) was applied to the dataset with nine extracted features of the hip joint angles for 10 subjects. The purpose of PCA is to visualize the class regions in the space of predictor variables (features) and to reduce the dimensionality of the data while retaining most of the variance present in the original features [56,57,58]. Based on the explained variance, the first three principal components were selected for further analysis, as they collectively account for 99.86% of the total variance. Further details and the rationale for this selection are discussed in Section 4.3.

After the PCA representation was performed, we employed a variety of ML methods from an open-source data-mining tool called the Waikato Environment for Knowledge Analysis (WEKA) software, version 3.8.6, to classify the extracted features [59,60,61,62]. The classification methods were chosen from five well-known classification categories, namely, Bayesian, function, lazy, rule, and tree classifiers, and to train the models, we chose 16 various classifier algorithms, as detailed in Table 2. The results were evaluated using classification accuracy and receiver operating characteristic (ROC). Classification accuracy measures the overall correctness of a model. The ROC curve, along with its area under the curve, evaluates a classifier’s ability to distinguish between classes across various thresholds [63,64,65].

In the context of WEKA, Bayesian classifiers, such as Bayesian network (BayesNet) and naive Bayesian (NB), use Bayes’s theorem to generate probabilistic outputs. BayesNet represents a set of variables via a directed acyclic graph (DAG), where each variable is a random variable and the edges are the probabilistic dependencies of the variable. The NB uses the Bayesian theorem with strong (naive) assumptions among the extracted features [60,66,67]. Still, determining the optimal sample size for training data is a crucial factor for achieving accurate classification performance, as it enables a closer approximation of the true data distribution. Thus, a novel approach has been introduced recently to estimate the minimum training sample size required for a Bayes classifier, which is detailed in a recent study [68]. This method employs a proxy learning curve, providing a practical framework for researchers to gauge the quantity of data necessary for their models to perform effectively. In the realm of the Naive Bayes (NB) classifier, it is important to note the simplifying assumption of feature independence given the class label, which, despite its potential to misrepresent the feature interdependencies, often results in a robust baseline for classification tasks due to its computational efficiency and surprisingly effective performance in high-dimensional settings.

The function classifiers utilize neural network and regression procedures in their functions [61]. Function classifiers such as logistic regression, multilayer perceptron (MultiPerceptron), sequential minimal optimization (SMO), simple logistic, and classification via regression (ClassViRegression) employ mathematical functions to represent relationships among the data connections. The MultiPerceptron and logistic regression are non-parametric supervised classifications, but the multilayer perceptron can predict more complex features than logistic regression, which usually predicts binary outcomes [69]. SMO utilizes the support vector machine (SVM) algorithm in the training procedures, whereas simple regression is a condensed version of logistic regression. However, solving the problem with classification can be handy using regression.

Lazy classifiers such as KStar calculate the distance between instances by employing a probabilistic measure based on the potential transformation of one instance into another, whereas the locally weighted learning (LWL) modifies the weight of each neighbor according to a distance function. However, the instance-based k (IBk) is the WEKA’s k-nearest neighbor (kNN) algorithm. All lazy classifiers defer model building until prediction time, making them efficient for certain datasets [70].

The rule classifiers such as Java repeated incremental pruning (JRip) implement repeated incremental pruning to produce an error reduction (RIPPER) algorithm [71]. The RIPPER builds a set of rules in the classification by repeatedly adding rules to the models to cover many instances and minimizing the error of overfitting [72]. A partial decision tree (PART) combines both decision trees with rule-based learning. Instead of building a full decision tree, it establishes rules by building and pruning partial decision trees; thus, it is called PART. PART utilizes partial C4.5 combined with the RIPPER algorithm in learning [73]. Both JRip and PART are known for producing models that are relatively easy to interpret.

Lastly, the tree classifiers were used, as they are among the most used classification techniques because of their ease of implementation [74]. Among the tree classifiers is J48, which implements the C4.5 algorithm developed by Ross Quinlan for generating decision trees from a set of training data and uses the concept of information entropy [75]. The other algorithm was the logistic model trees (LMTs) that build a decision tree based on simple class values at the leaves and based on a logistic regression model at each level of the tree (node). This LMT is used to capture the linear and non-linear instructions of the decision tree using logistic regression methods. Meanwhile, random forest (RF) can classify large amounts of data with accuracy, as it uses a multitude of decision trees and outputs the mode of classes at each individual tree. RF is robust for a large number of extracted features due to its capability to deal with overfitting [74]. However, for the fast decision tree learning procedure, a reduced error pruning tree (REPTree) was used. The algorithm builds a decision tree based on the gain/variance information to prune it using reduced error pruning with backfitting. REPTree uses the methods from C4.5 and the REP concept in its procedures [74]. Both RF and REPTree are known for their efficiency on large datasets [76].

## 4. Results and Discussion

### 4.1. Comparison of the Measuring Systems

**Figure 6:**
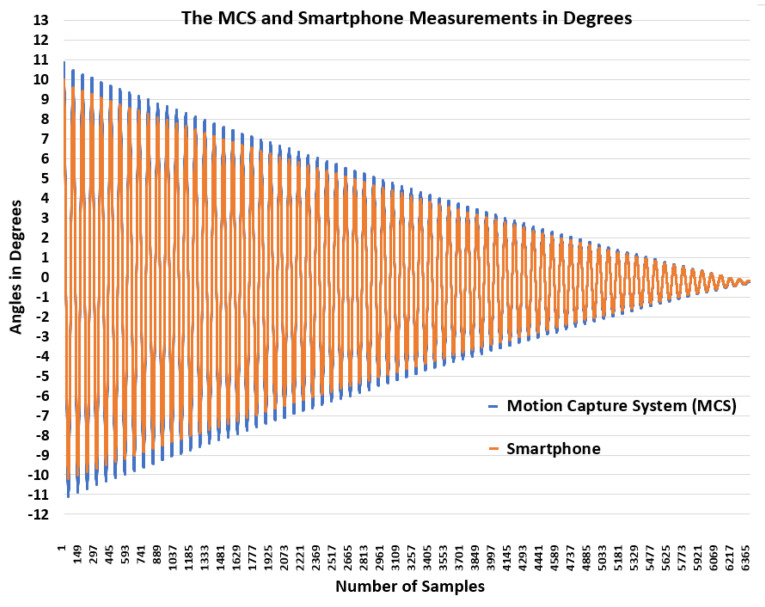
Comparison of measurements of MCS and smartphone in the pendulum test bench.

In this initial step before data acquisition, we examined the accuracy of measurements using smartphone sensors and motion-capture systems, benchmarked against a specially designed pendulum test bed. The focus was on assessing the precision and reliability of smartphone angle measurement compared with a well-known and precise measurement system represented by the MCS, thereby establishing confidence in smartphone measurements utilizing sensor fusion algorithms. The results are shown in Figure 6.

The obtained results reveal a high degree of congruence between the two systems across the oscillatory motion of the pendulum. Notably, the amplitude consistency and frequency alignment between the smartphone and MCS data were observed, with small differences in the amplitude of the two measurements. For this reason, the measurement performance of the smartphone compared with the MCS can be measured using the root mean square error (RMSE), as deduced by

(13) $$RMSE=\sqrt{\frac{\sum_{i=0}^{N}{\left(M_{\left(\mathrm{MCS}\right)\;}-M_{\left(\mathrm{smartphone}\right)\;}\right)}^{2}}{N}},$$

where *N* is the number of observation samples over time, which is, for these measurements, N=6500. The $M_{\left(\mathrm{MCS}\right)}$ represented in this work as the reference measurement using the MCS and $M_{\left(\mathrm{smartphone}\right)\;}$ is the measurement conducted by the smartphone.

Therefore, the calculated (RMSE = 0.34) indicates the efficacy of the smartphone in capturing the pendulum’s motion. Meanwhile, the attenuation in amplitude over time was consistent across both measurement modalities, suggesting a linear damping characteristic that is likely attributable to aerodynamic drag and mechanical friction at the pivot point. Furthermore, we noticed that the amplitude from the smartphone was less than that of the MCS, but did not affect the ability to identify subjects based on their gait patterns.

### 4.2. Hip Joint Angles

Through sensor fusion procedures in MATLAB, we transform raw data from accelerometer, gyroscope, and magnetometer sensors into hip joint angle measurements. The sensor fusion technique is detailed in the flowchart shown in Figure 5.

**Figure 7:**
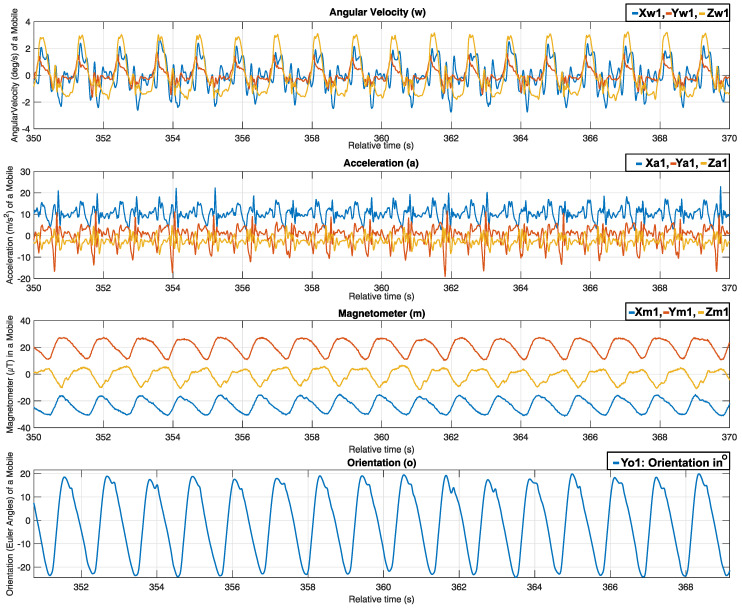
The acceleration, angular velocities, and magnetic field with the hip joint angle of a test subject.

This study pays special attention to movements within the sagittal plane, underpinning its relevance to rehabilitation scenarios. Therefore, we incorporate the significance of the pitch angle, outputted by the AHRS algorithm, as shown in Figure 2. The acceleration measurement, angular velocity, and magnetic field that are involved in the sensor fusion of a subject under the experimental test can be seen in Figure 7, as well as their results from the sensor fusion algorithm representing the hip joint angles being tested.

**Figure 8:**
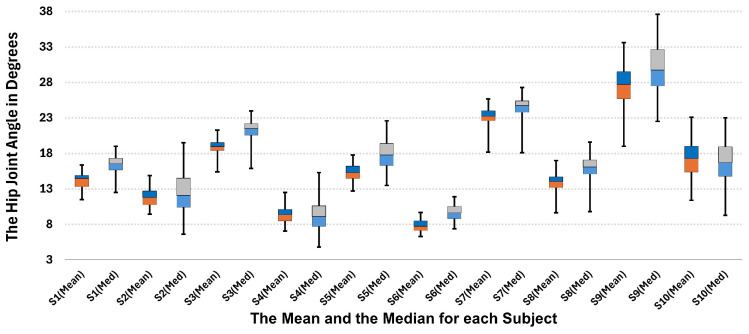
The boxplot for the mean value and the median features for all the experimental subjects.

Although the graph shows 20 s, it does not need to show huge differences within the steps as the subject walked on a treadmill, with a constant speed and floor (controlled environment); thus, we expect not to see huge differences in a human walking style within 20 s. However, the measurement data showed that there were differences in hip joint angles among the 10 subjects (people have various walking styles) and slight changes (a few degrees) within the subject steps when a subject became tired [53]. Within this context, some of these features extracted from the hip joint angle, such as the mean value and the median, for all the subjects under the experiment are shown in Figure 8.

### 4.3. Classification Analysis

Principal component analysis (PCA) was applied to the dataset with nine features to visualize their relationships, reduce the dimensionality of the data, and capture the variance in the data. For our study, we initially considered all nine components, as each component captures a part of the total variance. However, after analyzing the explained variance that is shown in Table 3, we determined that the first three principal components collectively account for 99.86% of the total variance, which is substantial and sufficient for our analysis. Therefore, we decided to use the first three PCA components for visualization and further analysis, ensuring that most of the information is retained while simplifying the dataset by reducing the dimensionality from nine to three.

**Figure 9:**
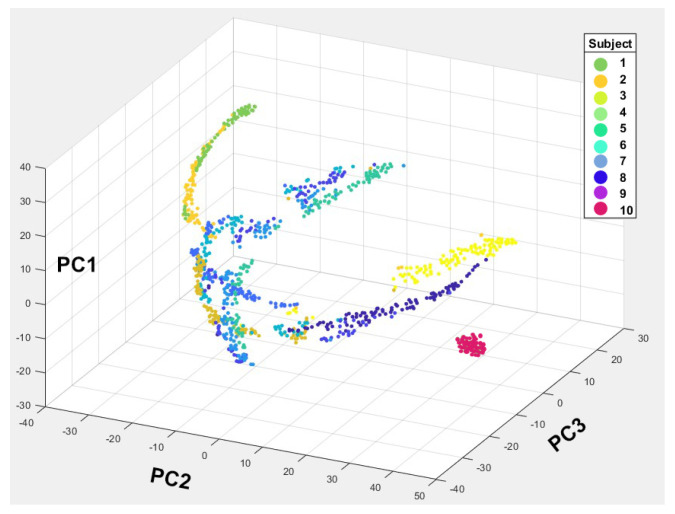
The 3D principal component analysis (PCA) for all subjects with color coding to differentiate each subject.

The choice of using the first three components is visually supported by the 3D PCA plot in Figure 9, where each data point represents a feature vector and each color represents a subject. The three axes (PC1,PC2,PC3) represent the directions of maximum variance in the data. PC1 accounts for the most variance, followed by PC2 and then PC3. The different colors represent the 10 different subjects. Each subject, demarcated by a unique color, presents a cluster formed by the data points, which indicate distinct separation between subjects’ gait patterns, demonstrating the efficacy of the dimensionality reduction. However, the proximal clustering within each subject suggests a high intra-subject consistency in gait features, while the spatial segregation between subjects underscores the inter-subject variability. Therefore, if the clusters are well separated, it suggests that the PCA has done a good job of distinguishing between the different subjects’ gait patterns. Additionally, if any points are far away from the main clusters, then they could be considered outliers. For this end, we do not see any significant outliers, which suggests that the gait patterns are relatively consistent within each subject.

The trajectories formed by the points (from one end of the graph to the other) can indicate the progression of the gait cycle for each subject. However, subjects 1, 3, and 9 form tight clusters, indicative of consistent and stable gait patterns with little variation. Subjects 2, 4, and 5 have more spread along the principal component axes, which may imply variability in specific gait characteristics. However, the spread is controlled, suggesting that these variations are systematic and could be related to individual walking styles or physiological differences. Subject 6 shows a distinct distribution, potentially indicating unique gait features that may differ significantly from the other subjects, while subjects 7 and 8 show a spread in the PCA space that suggests unique gait patterns. These subjects may have gait features that are less common among the cohort, which could be indicative of unique biomechanical traits. Lastly, subject 10 has its data points isolated from the rest, particularly along PC3. Such separation suggests that this subject has a gait pattern with distinct characteristics that are not shared with the other subjects, which could be of particular interest for specific gait analysis.

Five machine learning classifiers—16 algorithms—were trained and tested on hip joint angles based on nine extracted features. The data were trained by running each algorithm 10 times and choosing cross-validation with 10 folds in WEKA. It involves splitting the data into 10 subsets, with 1 subset used for testing and the rest for training in each iteration. Additionally, stratified cross-validation is used to maintain class distribution. The final estimate is an average of the 10 iterations, with an optional standard deviation. Ten-fold is preferred due to its proven accuracy and theoretical support. Repeated stratified cross-validation further enhances reliability.

When investigating classification accuracy (CA) and other metrics for various classification algorithms, certain tuning parameters were adjusted within the WEKA environment. WEKA’s graphical user interface provides a user-friendly platform for this purpose. For example, in the case of BayesNet, a combination of the SimpleEstimator with an alpha range of 0.5–0.8 and the LAGDHillClimber algorithm was used. This particular configuration showed superior CA compared with alternatives such as K2, SimulatedAnnealing, and TAN, which are also available in WEKA. As for the Naive Bayes (NB) classifier, the experiments were performed by switching the KernalEstimator between true and false while keeping the other WEKA default parameters constant. The MultiPerceptron was another interesting algorithm that was tested with both 5 and 6 hidden layers. To do this, we switched back and forth between ‘a’ and ‘t’ in the WEKA object editor. The sequential minimal optimization (SMO) algorithm was configured with the PolyKernel. This configuration resulted in the highest CA compared with other kernels such as Puk, StringKernel, and RBFKernel, all with their default parameters. Other classifiers such as SimpleLogistic, classifierViaRegression, KStar, PART, Logistic-R, and LMT were run with their default parameters in WEKA.

Local Weighted Learning (LWL) was selected with its default settings, but the LinearNNSearch algorithm was preferred over other options such as KDTree, Cover, and BalTree due to its higher CA. The instance-based K-nearest neighbor (IBK) classifier was tested with a KNN value of 9, which outperformed the other KNN values from 1 to 13 in terms of CA. JRip was run with an option of 9 folds for pruning, as this was found to be more effective compared with the default value of 1 fold. The J48 classifier was utilized with a confidence factor of 0.25, which is the default setting. Adjusting this value resulted in no significant differences in CA. The random forest (RF) classifier was selected with certain parameters: MaxDepth was set to 0, the number of trees in the forest to 100, and numFeature to 0, which determines the number of randomly selected attributes. Finally, REPTree was selected with its default settings, including a minNum of 2.0, which refers to the minimum total weight of instances in a leaf. Additionally, numFolds was set to 3, and maxDepth was set to −1, indicating no restrictions on the tree depth. This systematic approach to tuning the parameters and selecting the algorithms was crucial for optimal classification performance.

The evaluation of these algorithms was conducted based on several key metrics, such as CA, receiver operating characteristic (ROC), and classification interval (CI). The ROC area is a single measure of the overall performance of a classification model, with a higher area under the curve (AUC) value indicating better model performance—with values ranging from 0 to 1, where 0.5 denotes random guessing, and 1 signifies perfect performance. A CI serves as a quantitative measure of uncertainty in estimation, wherein the interval’s width is inversely related to the level of certainty. A broader confidence interval signifies a higher degree of uncertainty, whereas a narrower interval suggests increased confidence in the estimation [77]. To calculate the lower and upper limits *p* of the CI, we used a Wilson score interval method using Equation (14) [78,79].

(14) $$p=\left(f+\frac{z^{2}}{2N}\pm z\sqrt{\frac{f}{N}-\frac{f^{2}}{N}+\frac{z^{2}}{4N^{2}}}\right)/\left(1+\frac{z^{2}}{N}\right)$$

**Figure 10:**
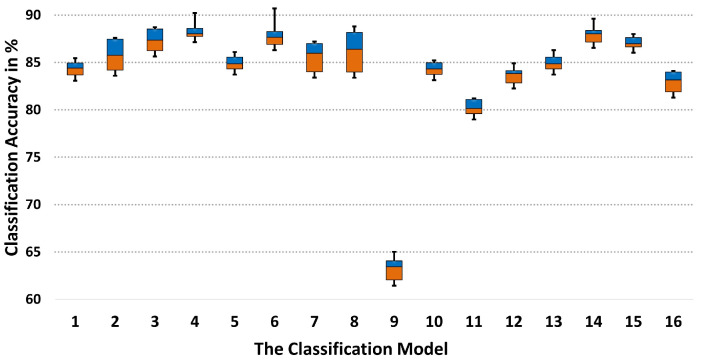
The boxplot of the classification accuracy across multiple models.

Here, *N* is the number of instances in the test set; *f* is the observed sample proportion (f=S/N), where *S* is the number of successes (or the number of correct guesses made by the model); and *z* is the *z*-score corresponding to the desired confidence level. In our study, when we use a confidence interval of 80% (with a wider interval indicating more uncertainty and a narrower one indicating higher confidence), *z* = 1.82. The term inside the square root is the adjusted standard error of the proportion, while the denominator is a correction factor that adjusts the interval’s width. The ± symbol indicates that the term comes after it adds for the upper limit of the confidence interval and subtracts for the lower limit. Therefore, the detailed CA, ROC, and CI of all the classification models by running the algorithms 10 times in WEKA can be seen in Table 4. Additionally, a boxplot in Figure 10 provides a graphical comparison among the classification models.

Table 4 and Figure 10 provide insight into the predictive capabilities of the machine learning models. The MultiPerceptron algorithm exhibited the highest classification accuracy (CA), indicating its effectiveness in handling the complex relationships within the gait data. Other models, like SimpleLogistic and LMT, also showed high accuracy and receiver operating characteristic (ROC) values.

**Figure 11:**
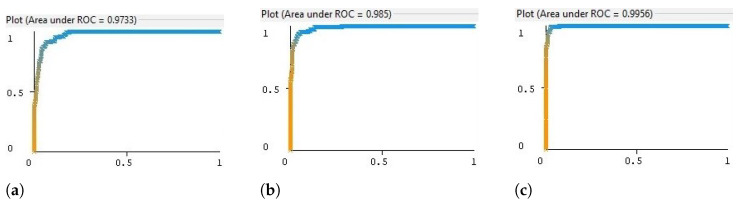
The ROC curve of the optimal classifier with the highest classification accuracy across different subjects: (**a**) the MultiPerceptron classifier for subject 2, (**b**) the SimpleLogistic classifier for subject 4, and (**c**) the LMT classifier for subject 6.

The evaluation metrics, such as CA, ROC, and classification intervals (CsI), served as critical indicators of model performance. The LMT algorithm demonstrated high CA and ROC, suggesting its strength in class probability estimation. The CI calculations provided by the equation helped quantify the uncertainty in model estimates, offering a comprehensive assessment of model reliability. These results suggest that a combination of PCA for feature reduction and a suitable selection of classifiers can yield robust and reliable insights into gait analysis for both research and clinical practice. For visualization purposes, the ROC areas for three subjects within the highest CA (MultiPerceptron, SimpleLogistic, and LMT) are shown in Figure 11.

The ROC curves show that the false positive rate (FPR,1-specificity) approaches zero, while the true positive rates (TPRs) for the respective classifiers are still quite high, indicating a strong performance in the low false alarm regime. The curves start at the top-left corner, which suggests that the classifiers can identify a significant number of true positives without incurring many false positives. However, as the FPR increases, the rate at which the TPR increases will differ among the classifiers. A steep initial slope in this region is desirable as it indicates that the classifier can achieve a high TPR without significantly increasing the FPR. Additionally, it is also crucial to consider the area under the ROC curve (AUC) values. The closer the AUC is to 1, the better the classifier’s overall ability is to distinguish between the positive and negative classes across all thresholds. The AUC values for the classifiers are substantially high (0.973, 0.985, and 0.995), indicating good overall performance.

These curves suggest that the classifiers perform well, particularly at low false alarm rates, which is often a critical area in many applications where the cost of a false alarm is high. By dissecting these results, we provide a nuanced understanding of each algorithm’s performance, offering valuable insights into their potential application in similar research contexts. These results can be a guide for future algorithmic choices in similar studies regarding their classification accuracy.

**Figure 12:**
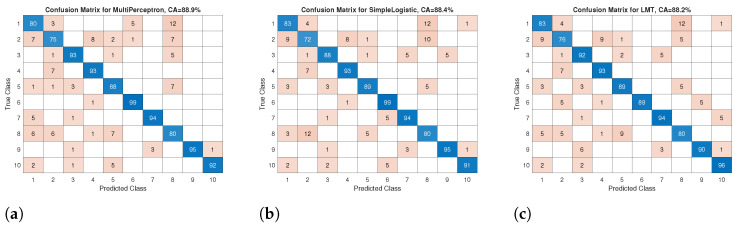
The confusion matrices for the classifier with the highest classification accuracy (the classes are from 1 = subject 1 to 10 = subject 10): (**a**) the confusion matrix for the MultiPerceptron classifier, (**b**) the confusion matrix for the SimpleLogistic classifier, and (**c**) the confusion matrix for the LMT classifier.

Additionally, the confusion matrices for the three classifiers (MultiPerceptron, SimpleLogistic, and LMT) with the highest classification accuracy (close to 89%) are depicted in Figure 12. The confusion matrices show that class 2 has variability in CA with the most confusion in distinguishing classes 1, 4 and 8 for the three classifiers. The same occurs with class 8, which shows confusion in classes 1, 2 and 5 with (12 instances) in SimpleLogistic. However, the three classifiers show sufficient CA to distinguish the various classes.

Furthermore, our study provides several contributions to smartphone applications and gait recognition by demonstrating the effectiveness of various classification algorithms for human recognition based on hip joint angles. However, other studies of gait recognition using sensors on ankle or wrist joints have shown promising results in gait analysis. For instance, Talha et al. [14] utilized a smartphone motion sensor on an ankle joint, achieving a classification accuracy of 87% with IMU raw data and 94% when using the gender and height feature in a training set. Similarly, Deb et al. [11] employed a time-warped similarity metric with accelerometer sensor data on wrist and ankle joints, resulting in a classification accuracy of 89.7% and 82.3%, respectively.

Our study utilizes conventional classifiers available in WEKA for their balance between performance and computational efficiency. Therefore, future research aims to explore advanced classifiers like LSTM NN, RNN, and HCNN to improve performance and capture complex gait patterns, building upon the solid foundation established by our conventional machine learning approaches. However, implementing these modern classifiers would require substantial changes to our setup, including deep learning frameworks like TensorFlow or PyTorch, which involve high computational demands and are beyond our current scope in this study.

Another future research direction may utilize our methods with diverse demographics of participants who have various hip or walking pathologies to compare the classification effectiveness with our current results. Further optimization of parameters and settings is worth investigating and could achieve the best possible performance and accuracy for the classification task. Therefore, we intend to investigate the integration of late fusion techniques, such as score fusion and majority voting strategies, to improve the accuracy and stability of the proposed models [91].

## 5. Conclusions

Our study investigates the utilization of smartphone IMU sensors to discriminate subjects based on their walking styles by analyzing hip joint angles. Our findings confirm the reliability of these sensors in measuring hip joint angles and effectively distinguishing between individuals using classification techniques. Through sensor fusion, which integrates accelerometer, gyroscope, and magnetometer data, we have achieved accuracy levels comparable with a reference system of angle measurements obtained from a camera array. By employing statistical methodologies for feature extraction and machine learning algorithms, we achieve an 88.9% classification accuracy. This underscores the immense potential of smartphones in facilitating comprehensive human walking analysis and proficiently classifying sensor data.

## Figures and Tables

**Table 1:** The mathematical representations and descriptions of the features [40,55].

Feature	Description	Mathematical Definition
Mean Value (MV)	The average of all angles in the sequence.	$\frac{1}{N}\sum_{i=1}^{N}x_{i}$
Median (M)	The middle angle value in the ordered sequence.	$M_{\mathrm{odd}}=x_{\left(\frac{N+1}{2}\right)},M_{\mathrm{even}}=\frac{x_{\left(\frac{N}{2}\right)}+x_{\left(\frac{N}{2}+1\right)}}{2}$
Maximum Angle	The largest angle observed.	$x_{\max}$
Covariance (COV)	Indicates how two angle variables vary together.	$\frac{1}{N-1}\sum_{i=1}^{N}\left(X_{i}-\bar{X}\right)\left(Y_{i}-\bar{Y}\right)$
Minimum Angle	The smallest angle observed.	$x_{\min}$
Variance (VAR)	Measures the dispersion around the mean angle.	$\frac{1}{N-1}\sum_{i=1}^{N}x_{i}^{2}$
Standard Deviation (SD)	Shows the amount of variation or dispersion of angle values.	$\sqrt{\frac{1}{N-1}\sum_{i=1}^{N}{\left(x_{i}-\bar{x}\right)}^{2}}$
Kurtosis (KUR)	Describes the sharpness or flatness of the angle distribution.	$\frac{1}{N}\sum_{i=1}^{N}{\left(\frac{x_{i}-\bar{x}}{\sigma }\right)}^{4}$
Skewness (SKE)	Shows the asymmetry in the angle distribution.	$\frac{1}{N}\sum_{i=1}^{N}{\left(\frac{x_{i}-\bar{x}}{\sigma }\right)}^{3}$

*N*: number of samples for each trial.

**Table 2:** The classification algorithms in WEKA.

No.	Category	Algorithms
1	Bayesian Classifiers	BayesNet
		NB
2	Function Classifiers	Logistic-R
		MultiPerceptron
		SMO
		Simple Logistic
		ClassViRegression
3	Lazy Classifiers	KStar
		LWL
		IBk
4	Rule Classifiers	JRip
		PART
5	Tree Classifiers	J48
		LMT
		RF
		REPTree

**Table 3:** Explained variance of principal components.

Principal Component	1	2	3	4	5	6	7	8	9
Explained Variance (%)	91.1337	8.4542	0.2717	0.1325	0.0073	0.0005	0.0001	0.0000	0.0000

**Table 4:** The classification accuracy of the multiple classifiers with rows shaded in gray indicating the highest classification accuracy (CA) percentage values for 10 subjects’ data [55].

No.	Classification Model	CA %	Av. ROC	Av. CI
1	BayesNet [80]	84.26 ± 1.2	0.975 ± 0.002	[0.829 0.859]
2	NB [81]	85.5 ± 2.1	0.988 ± 0.003	[0.856 0.884]
3	Logistic-R [82]	87.1 ± 1.6	0.986 ± 0.001	[0.865 0.892]
4	MultiPerceptron [83]	88.9 ± 1.3	0.965 ± 0.003	[0.874 0.899]
5	SMO [84]	84.9 ± 1.2	0.976 ± 0.002	[0.847 0.875]
6	SimpleLogistic [85]	88.4 ± 2.3	0.989 ±0.002	[0.861 0.907]
7	ClassViRegression [82]	85.4 ± 1.8	0.985 ± 0.004	[0.847 0.875]
8	KStar [86]	86.1 ± 2.6	0.987 ± 0.003	[0.849 0.887]
9	LWL [87]	63.4 ± 1.6	0.937 ± 0.001	[0.622 0.661]
10	IBk [86]	84.1 ± 1.1	0.917 ± 0.001	[0.830 0.852]
11	JRip [86]	80.1 ± 1.1	0.947 ± 0.003	[0.786 0.818]
12	PART [88]	83.4 ± 1.5	0.932 ± 0.001	[0.819 0.849]
13	J48 [86]	84.9 ± 1.4	0.937 ± 0.005	[0.814 0.844]
14	LMT [74]	88.2 ± 1.4	0.989 ± 0.001	[0.868 0.896]
15	RF [89]	86.9 ± 1.1	0.903 ± 0.001	[0.863 0.889]
16	REPTree [90]	82.9 ± 1.2	0.960 ± 0.001	[0.813 0.843]

CA: classification accuracy; M: mean, SD: standard deviation; Av. CI: average classification interval; Av. ROC: average receiver operating characteristic. All values under CA are in the form M ± SD).

## Data Availability

The measurement data are available on request from the corresponding author. The data are not publicly available due to data privacy protection regulations.

## Abbreviations

The following abbreviations are used in this manuscript:

MCS	motion capture system
EMG	electromyography
ML	machine learning
SVM	support vector machine
NN	neural network
LSTM NN	long short-term memory neural network
RNN	recurrent neural network
NB	naive Bayes
LDA	linear discriminant analysis
HCNN	hybrid convolutional neural network
IMU	inertial measurement unit
MARG	magnetic, angular rate, and gravity
MEMS	micro-electro-mechanical system
SFA	sensor fusion algorithm
LCF	linear complementary filter
NCF	nonlinear complementary filter
LKF	linear Kalman filter
EKF	extended Kalman filter
CKF	complementary Kalman filter
SRUKF	square root unscented Kalman filter
SRCKF	Square Root Cubature Kalman Filter
AHRS	attitude heading reference system
SD	standard deviation
RMSE	root mean square error
MV	mean value
M	median
COV	covariance
VAR	variance
KUR	kurtosis
SKE	skewness
PCA	principal component analysis
WEKA	Waikato Environment for Knowledge Analysis
BayesNet	Bayesian network
NB	naive Bayesian
SMO	sequential minimal optimization
LWL	locally weighted learning
kNN	K-nearest neighbor
IBk	instance-based K
JRip	Java repeated incremental pruning
RIPPER	repeated incremental pruning to produce error reduction error reduction
PART	partial decision tree
LMT	logistic model tree
REPTree	reduced error pruning tree
CA	classification accuracy
CI	confidence interval
ROC	receiver operating characteristic
AUC	area under the curve
